# Beta-Thalassemia Presenting as Moyamoya Syndrome With a Review of Skeletal Manifestations

**DOI:** 10.7759/cureus.38372

**Published:** 2023-05-01

**Authors:** Aswathy Sunil, Baranitharan Sivarajakumar, Vijaya Kumari

**Affiliations:** 1 Department of Radiology, Osmania Medical College, Hyderabad, IND

**Keywords:** magnetic resonance angiography, hair-on-end appearance, erlenmeyer flask deformity, ivy sign, puff of smoke, moya moya syndrome, beta-thalassemia

## Abstract

Moyamoya angiopathy (MMA) is a progressive vasculopathy characterized by slowly progressive stenosis involving the proximal portions of the major intracranial arteries, resulting in strokes and intracranial hemorrhages. If it occurs secondary to a known cause, it is called Moyamoya syndrome (MMS). Here, we describe the case of a five-year-old male child who presented to us with symptoms of stroke and, upon evaluation, revealed Moyamoya angiopathy. He was further evaluated, and it was found that MMA occurred as a complication of undetected beta-thalassemia. Thalassemia is an autosomal recessive blood disorder where there is a defect in hemoglobin production. It affects 100 to 150 thousand children in the Indian subcontinent. It is classified into two main types: alpha thalassemia and beta thalassemia, depending on which globin chain is affected. It primarily presents with symptoms of anemia such as easy fatiguability, dizziness, jaundice, or breathlessness. The occurrence of Moyamoya syndrome in beta-thalassemia is extremely rare, and it is extremely important to identify MMS at the earliest as it can cause long-term disabilities. We describe the imaging findings in MMS and the various classical skeletal radiographic findings in thalassemia that were seen in our patient.

## Introduction

MMA is a progressive vasculopathy characterized by slowly progressive stenosis involving the proximal portions of the major intracranial arteries. Collateral vessels (most commonly the lenticulo striate and thalamo perforator arteries) hypertrophy to compensate for the slowly progressive stenoses. These hypertrophied collaterals resemble a "puff of smoke" on angiography, an appearance from which the disease derives its name [1,2]. If MMA occurs due to any secondary cause, the disorder is termed Moyamoya syndrome (MMS) [3]. The common causes of MMS are neurofibromatosis type 1, trisomy 21, sickle cell anemia, radiation therapy, meningitis, etc. [1]. Thalassemia is a rarely described cause of MMS. We describe a case of MMS that occurred as a complication of undetected beta-thalassemia.

Thalassemia is a genetic blood disorder that affects the production of hemoglobin and is caused by mutations in the genes that control the production of hemoglobin. The severity of thalassemia can vary widely, from mild to severe, depending on the number of mutations and their effect on hemoglobin production. The disease hallmarks include an imbalance in the α/β-globin chain ratio, ineffective erythropoiesis, chronic hemolytic anemia, compensatory hemopoietic expansion, hypercoagulability, and increased intestinal iron absorption [4].

Thalassemia is a hypercoagulable state associated with the development of various thromboembolic phenomena. It can rarely result in MMA, with only a few cases reported [3,5,6]. Das et al. studied 75 cases of MMA over a span of three years and found the rare occurrence of thalassemia and MMA in four patients [7].

Radiologically, the complications of thalassemia can be detected through various imaging techniques, including X-ray, computed tomography (CT), and magnetic resonance imaging (MRI). In patients with severe thalassemia, there may be radiological evidence of bone deformities and bone marrow expansion as the body tries to compensate for the lack of healthy red blood cells. This can lead to abnormalities in the skull, facial bones, and long bones of the limbs [8].

## Case presentation

A five-year-old male child presented to the emergency department with chief complaints of sudden-onset left-sided hemiparesis and loss of consciousness. There was no history of fever, seizure, trauma, or recent infection, nor was there any prior history of focal neurological deficit (FND) or cognitive or psychiatric manifestations. A history of hospital admission was present four months ago for severe anemia, for which multiple blood transfusions were given.

On clinical examination, tachycardia and pallor were seen. Neurologic examination revealed left-sided diminished muscle power in the upper and lower limbs with spasticity, brisk deep tendon reflexes (DTR), and an extensor plantar response.

Haematological investigations

Blood counts were within normal limits. The initial hemoglobin level was found to be 5.2 gm%, suggesting severe anemia. A peripheral smear revealed microcytic and hypochromic anemia. The Sickling test was negative. High-performance liquid chromatography (HPLC) showed significantly increased HbF (95%) and HbA2 (6%), suggesting beta-thalassemia major. HPLC was done for parents and siblings, which identified them as having beta-thalassemia traits.

Radiological investigations

**Figure 1:**
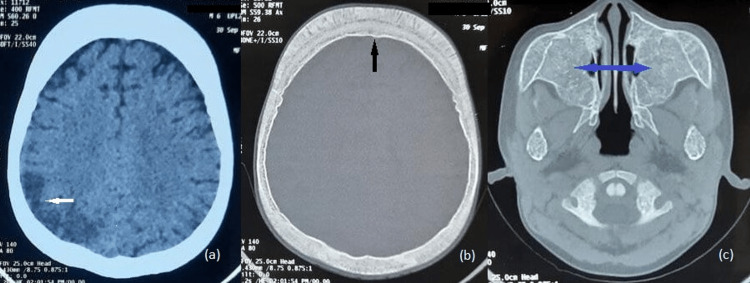
Non-enhanced axial CT brain. (a) Hypodensity in the right parietal cortex with loss of gray-white matter differentiation (white arrow). (b) Axial CT brain in the bone window showing thickened calvarium with widening of diploic space in the frontal bones (black arrow). (c) Absent pneumatization of bilateral maxillary sinuses (blue arrow).

The CT brain showed hypodensity involving the right parieto-temporal lobe with loss of gray-white matter differentiation. Calvarial and facial bone thickening with hypo-pneumatization of the paranasal sinuses was noted (Figure 1).

**Figure 2:**
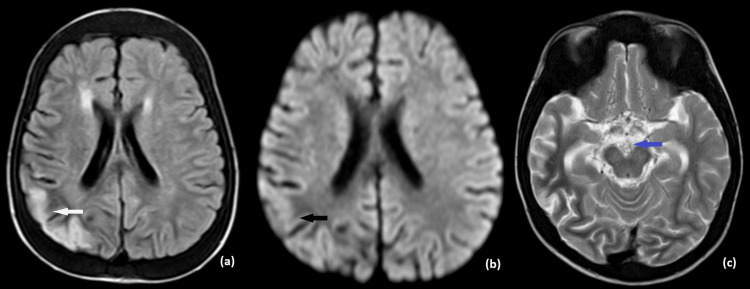
Axial sections of MRI brain. FLAIR (a) hyperintensity (white arrow) in the right parietal lobe with no restricted diffusion on DWI (b) (black arrow) suggestive of subacute infarct. Axial T2 weighted image (c) showing numerous vascular collateral channels in the basal cisterns (blue arrow).

**Figure 3:**
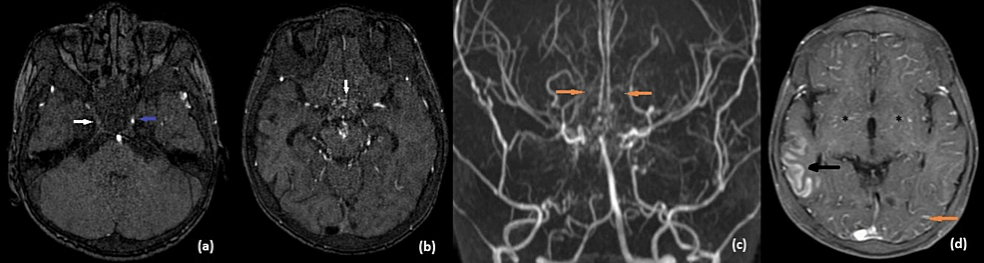
MR angiogram showing occlusion of right ICA from C3 segment (white arrow) and narrowed left ICA (blue arrow) (a) with multiple collateral vascular channels in the suprasellar cistern (white arrow) (b). Coronal maximum intensity projection (MIP) image (c) showing multiple collaterals giving the classical “puff of smoke” appearance (yellow arrows). Axial fat suppressed post contrast T1 weighted image (d) showing gyral enhancement pattern in the right temporal lobe (black arrow). Leptomeningeal enhancement seen due to prominent leptomeningeal collaterals giving an “ivy” appearance (yellow arrow). Multiple collaterals are seen in bilateral basal ganglia (asterisks).

Subsequent MRI of the brain with contrast revealed a subacute infarct of the right pareito-temporal lobe with post-contrast enhancement. Magnetic resonance angiography (MRA) depicted occlusion of the right internal carotid artery from the C3 segment and narrowing of the left internal carotid artery with multiple collateral formations in bilateral basal ganglia, giving a "puff of smoke" appearance, suggestive of Moya Moya angiopathy (MMA). In the post-contrast study, enhancement of leptomeningeal collaterals was present, giving the "ivy sign" (Figures 2-3).

**Figure 4:**
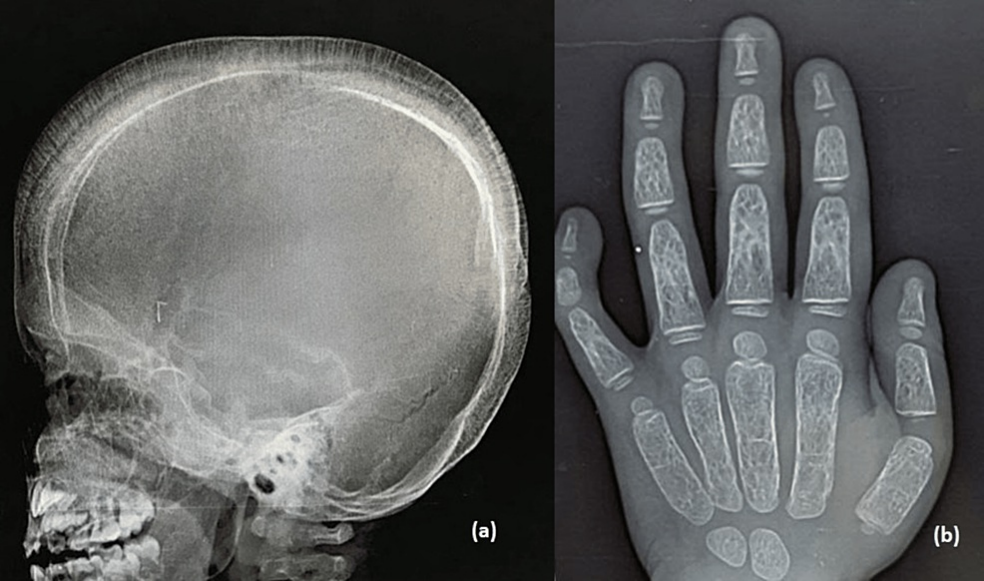
Lateral skull radiograph (a) showing widened diploic space with “hair-on-end” appearance and thinning of the outer table of the skull. Sparing of the occipital bone is seen (due to lack of hematopoietic bone marrow). AP hand radiograph (b) showing osteopenia, sausage-shaped tubular bones with cortical thinning, medullary expansion, and coarse trabeculation.

**Figure 5:**
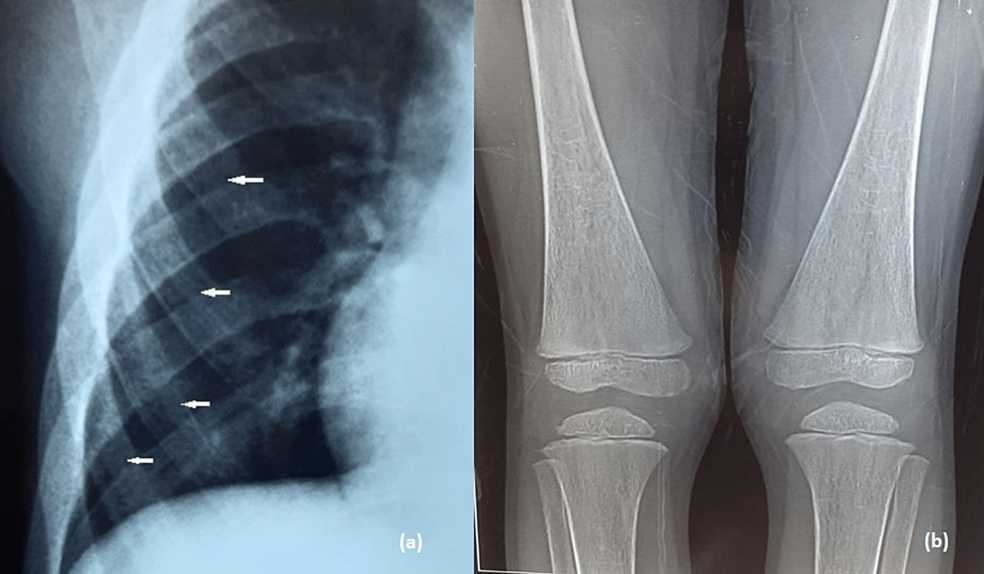
Magnified view of chest radiograph PA view (a) showing expansion of ribs with a “rib-within-a-rib” appearance, particularly in the anterior portion (white arrows). Knee radiograph (b) showing bilateral metaphyseal flaring with Erlenmeyer flask deformity.

The skull radiograph showed widened diploic space with a "hair-on-end" appearance (Figure 4a). The hand radiograph showed sausage-shaped tubular bones with thinning of the cortex, medullary expansion, and coarse trabeculation (Figure 4b). Chest radiographs revealed expansion of ribs with a "rib-within-a-rib" appearance (Figure 5a), and knee radiographs revealed metaphyseal flaring with Erlenmeyer flask deformity (Figure 5b).

## Discussion

Moyamoya disease (MMD) is a progressive vasculopathy leading to stenosis of the main intracranial arteries with the development of collateral vasculature that produces a typical angiographic image called "clouds of smoke" or "puffs of cigarette smoke." The disease is most common in children and young adolescents. There are observed two peaks of incidence: in the first and third-fourth decade of life [1,9]. The vessels of the collateral circulation are formed as a result of the widening of the existing vessels or the development of new perforating arteries. There are three main pathways of collateral circulation - parenchymal, meningeal, and transdural [9].

Idiopathic Moyamoya angiopathy is termed Moyamoya disease, while MMA secondary to an underlying cause is called Moyamoya syndrome [1,2]. The presentation of MMS is variable and includes transient ischemic attack (TIA), ischemic or hemorrhagic stroke, and epilepsy, although it can rarely develop dystonia, chorea, or dyskinesia. The imaging findings in Moyamoya disease are classified as primary and secondary. The primary findings essentially consist of the occlusion of the circle of Willis and collateral formation, including Moyamoya vessel formation. The secondary findings include cerebral infarction, white matter lesions, atrophy, and hemorrhage. Although digital subtraction angiography was earlier considered the gold standard for the diagnosis of MMD, MR and CT angiography can noninvasively demonstrate the stenotic or occlusive lesions in distal ICAs and the arteries around the circle of Willis, including the collateral Moyamoya vessels in the basal ganglia [9,10]. The classical findings include focal regions of acute infarction or localized or diffuse atrophy depending upon the site(s) of vascular narrowing and the adequacy of collateral flow, loss of the normal flow void in the distal carotid branches, an extensive tangle of collaterals that develop distal to occluded vessels ("puff of smoke"), and prominent leptomeningeal collaterals resulting in T2 FLAIR/post-contrast T1 sulcal/subarachnoid hyperintensities that resemble growing ivy ("ivy sign").

The common causes of MMS are neurofibromatosis type 1, trisomy 21, sickle cell anemia, radiation therapy, meningitis, etc. [1]. Beta-thalassemia is a rarely described cause of MMS [3,5-7]. Hemolysis and ineffective erythropoiesis together cause anemia in thalassemia, which may cause tissue hypoxia and hypertrophic vascular endothelium, leading to microvascular stenosis [11]. There is a hypercoagulable state in thalassemia that is multifactorial and attributed to endothelial activation, altered platelet function, red blood cell membrane abnormalities leading to activation of the coagulation cascades, and changes in coagulation protein levels [3]. Thromboembolic phenomena, both venous and arterial, are thus not uncommon in patients with thalassemia. Risk factors for thrombosis include a family history of a thrombotic event, previous splenectomy, profound anemia, infrequent transfusions, age above 35 years, and a serum ferritin level ≥1000 mg/l. On the other hand, a positive history of transfusion and a hemoglobin level ≥9 g/dl were found to be protective against thrombosis [7]. This hypercoagulable state can rarely result in the development of MMS.

The ineffective erythropoiesis in thalassemia results in marrow expansion, which affects both cortical and cancellous bones, causing widening of the medullary space, cortical thinning, and resorption of the secondary/tertiary bone trabeculae, with subsequent prominent/coarse primary trabeculae creating a "lace-like" appearance. Osteopenia/osteoporosis is seen [8,12].

A skeletal survey was done for our patient, which revealed various classical imaging findings of thalassemia, as described below.

Skull

Significant marrow hyperplasia causes the widening of the diploic space. Trabeculae become thickened and are vertically oriented to the inner table. Thinning of both tables occurs (outer>inner), with eventual perforation of the outer table. This results in a "crew-cut" or "hair-on-end" appearance [13].

Paranasal sinuses

Marrow hyperplasia occurring in the facial and calvarial bones results in hypopneumatization of the paranasal sinuses. The maxillary sinus is most commonly affected, with ethmoid air cells affected the least. Changes in the sinuses are a classical X-ray finding in βTM and are not usually observed in other anemias [8].

Ribs

Widening and expansion of ribs along their entire length, along with a central dense area, particularly in the mid and anterior portions. This appearance is called a "rib-within-a-rib" appearance [8].

Hand and foot

Diffuse osteopenia with medullary expansion and cortical thinning can be seen. Marrow hyperplasia causes the bulging of metacarpals, metatarsals, and phalanges, resulting in sausage-shaped bones. Another finding is an enlarged nutrient foramen [8].

Long bones

Marrow hyperplasia causes medullary widening and cortical thinning with increased trabeculation. Marrow hyperplasia also causes the loss of the normal concavity of the epi-metaphysis and makes them straight or even convex, resembling the Erlenmeyer flask deformity. Growth arrest lines/Harris lines may be seen at the metaphysis [8].

Spine

Diffuse osteoporosis can be seen. Initially, marrow hyperplasia can cause mild enlargement of vertebral bodies. However, eventually, multiple small compression fractures can develop, causing thinning of the subchondral bone and a biconcave vertebral deformity called a "fish-type" vertebra. Vertebral collapse and platyspondyly are seen [8].

## Conclusions

Beta-thalassemia is a hereditary anemia resulting from defects in hemoglobin production. The incidence of MMS in beta-thalassemia is rare, with only a few cases reported in the past. In most cases, the diagnosis of beta-thalassemia was made substantially earlier than the diagnosis of MMS. However, in our case, the child presented with a stroke due to MMS, and subsequent evaluation showed beta-thalassemia. MRI with MRA is an invaluable non-invasive technique for the diagnosis of MMS since its early detection is essential to avoiding disability.
